# Perioperative changes in IgG and plasma N-glycosylation in children with acute appendicitis and elective surgery: a prospective study

**DOI:** 10.3325/cmj.2026.67.164

**Published:** 2026-06

**Authors:** Jasna Obuljen, Nikol Mraz, Tea Pribić, Irena Trbojević-Akmačić, Irena Linarić, Jasna Leniček Krleža, Stjepan Višnjić, Marko Mesić, Mislav Bastić, Anto Pajić, Neda Striber, Anđelo Beletić, Zoran Bahtijarević, Gordan Lauc, Dragan Primorac, Borna Rapčan

**Affiliations:** 1 Department of Clinical Chemistry, Sestre Milosrdnice University Hospital Center, Zagreb, Croatia; 2 Genos, Zagreb, Croatia; 3 Department for Clinical Chemistry, Children's Hospital Zagreb, Zagreb, Croatia; 4 Department of Pediatric Surgery, Children's Hospital Zagreb, Zagreb, Croatia; 5 Polyclinic Synevo Croatia, Zagreb, Croatia; 6 Union of European Football Associations, Nyon, Switzerland; 7 Faculty of Pharmacy and Biochemistry, University of Zagreb, Croatia; 8 St. Catherine Specialty Hospital, Zabok/Zagreb, Croatia; 9 Eberly College of Science, The Pennsylvania State University, University Park, PA, USA; 10 University of Split, School of Medicine, Split, Croatia; 11 University of Osijek, School of Medicine, Osijek, Croatia

## Abstract

**Aim:**

To compare plasma protein and IgG N-glycosylation levels in pediatric patients undergoing acute appendicitis surgery and in those undergoing minor elective surgery. The secondary aim was to explore whether the plasma N-glycome profile may serve as a biomarker of systemic inflammatory burden in children.

**Methods:**

This prospective study enrolled children aged 4-16 years undergoing minor elective surgery (n = 38) or surgery for acute appendicitis (n = 19) at Children’s Hospital Zagreb from 2017 to 2021. Blood samples were collected preoperatively and 24 hours postoperatively. IgG and total plasma N-glycans were assessed using high-throughput hydrophilic interaction ultra-high-performance liquid chromatography. Derived glycan traits were compared between the groups and across timepoints.

**Results:**

Compared with elective surgery controls, children with acute appendicitis exhibited extensive plasma glycomic remodeling. This was characterized by increased branching, sialylation, and antennary fucosylation, along with reduced abundance of less complex glycans, indicating a systemic inflammatory glycan phenotype. IgG glycosylation also differed between the groups but showed fewer and less pronounced changes. Minor elective surgery, despite increased C-reactive protein levels, did not significantly alter either plasma or IgG glycomes. Twenty-four hours after surgery, children with appendicitis exhibited significant postoperative changes only in the plasma N-glycome, while IgG glycosylation remained stable.

**Conclusion:**

Plasma N-glycosylation reflects inflammatory burden rather than surgical stress. These findings highlight plasma glycome profiling as a potential systems-level biomarker for assessing acute inflammation and disease severity in pediatric populations.

Protein glycosylation represents an evolutionarily conserved, complex, highly specific, and tightly regulated process of co- and post-translational addition of oligosaccharide structures known as glycans (1). Depending on the atom through which glycans attach to the protein, we distinguish N-, O-, C-, S-, and P-glycosylation (phosphoglycosylation). N-glycosylation is the most prevalent and extensively studied form, in which the glycan is attached to the protein via the nitrogen atom in the side chain of asparagine (2).

An important structural feature of glycosylated proteins is glycan heterogeneity, which may manifest as differences in branching, presence or absence of particular monosaccharides (eg, sialic acid, N-acetylglucosamine, fucose), or differences in glycosidic linkages between monosaccharide units (3). More than half of all known proteins are glycosylated (4), and this modification influences their structure, processing, folding, half-life, and consequently their effector functions (5). In contrast to proteins, glycan biosynthesis is not template-driven; rather, glycan structures are shaped by the coordinated influence of the cellular milieu, glycosylation enzyme expression, and substrate supply (6). Glycosylation and glycans play indispensable roles in numerous physiological processes, including cell recognition, regulation of gene expression, intracellular protein trafficking, and immune system regulation (7-10).

Immunoglobulin G (IgG) is the most abundant and best-studied glycoprotein in blood plasma. IgG contains two conserved N-glycosylation sites in the Fc region, while 15-25% of plasma IgG carries attached glycans in the variable Fab regions as well (11). IgG effector functions are regulated by changes in Fc glycosylation, whereby galactosylation and sialylation exert anti-inflammatory effects, while their absence and the lack of core fucose promote enhanced inflammatory responses (12,13). Plasma protein and IgG glycan profiles vary considerably at the population level due to the effects of age, sex, lifestyle, and other genomic and environmental factors. However, at the individual level, the glycome is considered relatively stable in the absence of major pathophysiological changes (14-18). This stability has prompted studies investigating plasma and IgG glycome changes as disease biomarkers, while advances in high-throughput glycan analysis have improved analytical capacity and reproducibility (19). IgG glycosylation alterations have been extensively studied in rheumatoid arthritis, diabetes, metabolic syndrome, and other cardiometabolic, autoimmune, malignant, and infectious diseases (20-22).

The glycosylation of proteins in children has been less studied. A study of healthy children aged 6-18 years demonstrated a higher proportion of complex glycan structures (tri- and tetra-sialylated glycans) in the plasma N-glycome of younger children, with an age-associated increase in disialylated biantennary structures. Also, fucosylation and levels of agalactosylated plasma and IgG glycans decreased with age, while digalactosylated glycans increased; dynamics opposite to those observed in adults. Furthermore, sex-related differences are less pronounced than in adults and emerge primarily during puberty (23). In clinical disciplines, research on congenital disorders of glycosylation has the longest tradition (24), while over the past decade interest has expanded into other pediatric fields such as immunology, pulmonology, diabetology, and neurosurgery (25-27).

Acute and chronic inflammatory processes are accompanied by numerous molecular and cellular changes that manifest locally at the site of inflammation as well as systemically at biochemical and physiological levels (28). Current knowledge of inflammation-associated glycosylation primarily concerns chronic inflammation, while glycosylation changes during acute inflammation remain less studied due to the dynamic nature of the acute inflammatory response. Previous studies have described glycosylation changes in various plasma proteins, including α1-acid glycoprotein, IgG, IgA, transferrin, haptoglobin, and α2-macroglobulin (29). Glycosylation changes following surgery can predict individual responses to acute inflammation after cardiac (30) and major abdominal procedures (31). Additionally, altered transferrin sialylation has been identified as a predictor of acute pancreatitis severity (32) and as a potential novel biomarker of sepsis severity (33). However, data on glycosylation changes in the pediatric population remain scarce despite the fact that the developing immune system in children exhibits distinct functional and compositional characteristics compared to adults (34). This study therefore aimed to investigate plasma protein and IgG N-glycosylation changes in pediatric patients with varying degrees of acute inflammation, comparing children with acute appendicitis to those undergoing elective minor surgery. A secondary aim was to explore whether plasma N-glycome profiling may serve as a biomarker of systemic inflammatory burden in children.

## Participants and methods

### Study participants

Whole blood, plasma, and serum samples were collected from children aged 4-16 years hospitalized at the Children’s Hospital Zagreb from 2017 to 2021 for surgical procedures. Participants enrolled in this prospective study were divided into two groups: 1) children without prior inflammatory conditions undergoing minor elective surgical procedures (phimosis repair, orchidopexy for undescended testis, hernia repair, strabismus surgery) (N = 38) and 2) children undergoing surgery for acute appendicitis (N = 19).

The clinical diagnosis of appendicitis and the indication for surgery were established based on clinical signs, abdominal ultrasound findings, and elevated laboratory markers of acute systemic inflammation. Inclusion criteria for the appendicitis group included phlegmonous, gangrenous, or perforated appendicitis.

The presence and intensity of systemic inflammation before and after surgery were assessed based on C-reactive protein (CRP) concentrations. CRP levels were interpreted as follows: ≤5 mg/L – an absence of inflammation; 5-20 mg/L – low-grade inflammation; 20-75 mg/L – moderate inflammation; and >75 mg/L – severe inflammation. Categories were adapted from ranges reported in the literature and adjusted to suit the clinical context of this study (35,36).

Blood samples were collected twice (before surgery and 24 hours postoperatively) into two vacuum tubes. Blood collected in tubes without anticoagulant was allowed to clot for 30 minutes and then centrifuged for 10 minutes at 1500 × g to separate serum for CRP measurement. Blood collected in K2EDTA tubes (Vacuette blood collection tubes, Greiner Bio-One, Kremsmünster, Austria) was centrifuged under identical conditions. Plasma was separated and stored at -80°C until glycome analysis.

The study aligned with the principles of the Declaration of Helsinki and was approved by the Ethics Committee of the Children’s Hospital Zagreb, Zagreb, Croatia (01-26/19-14) and the Ethics Committee of the Faculty of Pharmacy and Biochemistry, University of Zagreb (004-01/24-03/01). Written informed consent was obtained from parents or legal guardians, as well as from children older than 7 years, in accordance with the Ethics Committee approvals.

### Isolation of IgG from human plasma

Prior to IgG isolation, all samples were randomized across 96-well plates, and 10 μL of plasma was aliquoted for total plasma protein N-glycan analysis. IgG was isolated from plasma using CIM® r-Protein G LLD 0.2 mL Monolithic 96-well Plate (2 µm channels) (Sartorius BIA Separations, Ajdovšćina, Slovenia) as previously described (37). Briefly, 100 μL of plasma was filtered through an AcroPrep Advance wwPTFE 1 mL, 0.45 μm 96-well filter plate (Citiva, New York, NY, USA), diluted with 700 μL of 1× phosphate-buffered saline (PBS, prepared in-house), and applied to a 96-well monolithic plate. The columns were washed repeatedly with 2 mL of 1× PBS to remove unbound proteins. Bound IgG was eluted using 1 mL of 0.1 M formic acid (Sigma-Aldrich, St. Louis, MO, USA), and the eluate was immediately neutralized with 170 μL of 1 M ammonium bicarbonate (Sigma-Aldrich). 300 µL of isolated IgG was dried in a vacuum centrifuge and stored at -20 °C until further analysis of released N-glycans.

### Enzymatic release and fluorescent labeling of N-glycans from IgG and total plasma proteins

High-throughput profiling of IgG and total plasma protein N-glycans was performed using an ultra-high-performance liquid chromatography based on hydrophilic interactions (HILIC-UHPLC) (37,38).

Dried IgG samples were resuspended in 30 μL of 1.33% SDS (Sigma-Aldrich) and denatured at 65°C for 10 minutes. Plasma samples were denatured by adding 20 μL of 2% SDS followed by incubation at 65°C for 10 minutes. Subsequent steps were identical for both IgG and total plasma protein samples.

Following denaturation, 10 μL of 4% Igepal-CA630 (Sigma-Aldrich) was added, and samples were incubated at room temperature with shaking for 15 minutes. N-glycans were enzymatically released by adding 1.2 U of PNGase F (Promega, Madison, WI, USA) and incubating at 37°C for 18 hours.

Released glycans were fluorescently labeled with 2-aminobenzamide (2-AB) via reductive amination. The labeling mixture consisted of 0.48 mg 2-AB and 1.12 mg 2-picoline borane (both Sigma-Aldrich) dissolved in 25 μL of 30% (v/v) acetic acid (Merck, Darmstadt, Germany) in dimethyl sulfoxide (Sigma-Aldrich). The reaction was carried out at 65°C for 2 hours.

Labeled glycans were purified by solid-phase extraction. Briefly, 700 μL of cold acetonitrile (ACN; VWR International, Radnor, PA, USA) was added to each sample, and the mixture was transferred to a 96-well 1-mL AcroPrep wwPTFE 0.2 μm filter plate (Cytiva). Solvents were removed under vacuum, and samples were washed five times with 96% ACN. The labeled N-glycans were eluted with 2× 90 μL of ultrapure water, and the combined volume was stored at -20°C until chromatographic analysis.

### Hydrophilic interaction liquid chromatography of labeled N-glycans

Fluorescently labeled N-glycans were separated by HILIC-UHPLC using an Acquity UPLC H-Class system (Waters, Milford, MA, USA). Fluorescence detection was performed with excitation and emission wavelengths set at 250 nm and 428 nm, respectively.

N-glycans were separated using ACQUITY PREMIER Glycan BEH Amide, 130Å, 1.7 µm columns (Waters), 2.1 × 150 mm for plasma and 2.1 × 100 mm for IgG analyses. Solvent A consisted of 100 mM ammonium formate in water (pH 4.4), and solvent B was 100% ACN.

Plasma N-glycans were separated using a linear gradient of 30-47% solvent A over 25 minutes at a flow rate of 0.56 mL/min. IgG N-glycans were separated using a 25-38% linear gradient of solvent A over 29 minutes at a flow rate of 0.4 mL/min.

Chromatographic data were initially processed using an automated integration method, followed by manual inspection and correction to ensure consistent peak integration across samples. Chromatograms were divided into 24 peaks (IGP1-IGP24) for IgG N-glycans and 39 peaks (GP1-GP39) for plasma N-glycans. The glycan composition corresponding to each peak had been previously validated by LC-MS analysis (14,39). Representative chromatograms and major glycan structures for total plasma and IgG N-glycans are presented in Supplemental Figure 1(Supplementary Figure 1) and Supplemental Figure 2(Supplementary Figure 2).

### Statistical analysis

Data are presented as medians (minimum-maximum). Differences between children undergoing elective surgery and children with acute appendicitis at each time point (preoperative and 24 hours postoperative) were assessed using the Mann-Whitney U test. Within-group comparisons between preoperative and postoperative measurements were performed using the Wilcoxon signed-rank test for paired samples. Analyses were conducted separately for IgG N-glycan traits and total plasma protein N-glycan traits.

To control for multiple testing and reduce the risk of false-positive findings, the Benjamini-Hochberg false discovery rate (FDR) correction was applied. All statistical tests were two-sided, and adjusted p-values < 0.05 were considered statistically significant. Statistical analysis was performed using Python (version 3.14.0). Data were preprocessed using pandas (version 2.3.3), and results were exported using the xlsxwriter package (version 3.2.9). Statistical testing was performed using SciPy (version 1.16.3). Data visualization was carried out using matplotlib (version 3.10.7) and seaborn (version 0.13.2).

## Results

The median age (minimum-maximum) of healthy children admitted for minor surgery was 7 (5.5-10.0) years (n = 38), while that of children with appendicitis was 9.5 (4.0-16.0) years (n = 20). The male-to-female ratio was comparable between the groups: 31/7 in the elective surgery group and 13/7 in the appendicitis group. CRP levels in children undergoing minor surgery increased from a median of 0.3 mg/L preoperatively to 3.3 mg/L postoperatively. In children with appendicitis, markedly elevated preoperative CRP levels decreased following surgery but remained high (175.7 mg/L preoperatively vs 164.5 mg/L postoperatively).

A total of 31 out of 39 glycan peaks significantly differed between the groups preoperatively (Table 1). Children undergoing minor surgery had a higher relative abundance of early eluting peaks (GP1-GP13), representing smaller and less complex glycans. Regarding glycans of intermediate structural complexity (GP14-GP23), several peaks (including GP14, GP20, and GP21) were higher in the appendicitis group, whereas others remained higher in healthy children. Notably, GP20 (A2G2S2), the dominant plasma glycan peak, showed higher abundance in children with appendicitis. Among the most complex glycans (GP24-GP39), a shift toward more highly branched and structurally elaborated species was evident, with multiple peaks increased in the appendicitis group, although not uniformly across all complex structures.

**Table 1:** Plasma glycan peaks (GP1-GP39) and 16 derived glycosylation traits in children undergoing elective surgery and children with appendicitis. Values represent median normalized abundances for each group

GP number	Minor elective surgery	Appendicitis group	p	q
1	3.4369	2.5734	0.0360	0.0439
2	1.5593	1.1066	0.0001	0.0002
3	0.0311	0.0242	0.0151	0.0192
4	3.7972	2.8384	0.0028	0.0042
5	1.5087	1.0838	0.0265	0.0329
6	0.7907	0.6375	0.0092	0.0122
7	0.9551	0.7331	4.5240 × 10^−5^	0.0001
8	1.2665	1.2713	0.3570	0.3999
9	0.0704	0.0511	0.0004	0.0008
10	4.1186	2.8379	2.0962 × 10^−5^	0.0001
11	0.5148	0.4375	0.0393	0.0468
12	1.4918	1.1401	6.5692 × 10^−7^	4.0268 × 10^−6^
13	0.5990	0.3899	0.0102	0.0132
14	13.0784	13.3773	0.8401	0.8694
15	0.3163	0.3005	0.8538	0.8694
16	5.4647	3.8598	2.6171 × 10^−7^	1.8320 × 10^−6^
17	1.2317	0.9751	0.0064	0.0087
18	4.4100	3.7507	4.8779 × 10^−5^	0.0001
19	1.0269	0.8015	5.1121 × 10^−8^	8.4735 × 10^−7^
20	28.4961	34.5047	0.0001	0.0003
21	0.5198	0.5512	0.1164	0.1330
22	3.8171	3.2426	0.0023	0.0037
23	1.1921	0.8720	2.6485 × 10^−5^	0.0001
24	1.6800	1.4507	0.0031	0.0046
25	0.3243	0.3745	0.0694	0.0810
26	1.5917	1.6197	0.5219	0.5731
27	0.6433	0.9988	0.0003	0.0006
28	0.7910	0.5653	3.7858 × 10^−6^	1.7667 × 10^−5^
29	0.2272	0.3728	0.0003	0.0006
30	5.2749	5.2734	0.5334	0.5744
31	0.4562	0.4448	0.8953	0.8953
32	1.3146	1.8245	1.6547 × 10^−5^	0.0001
33	2.2820	3.4360	0.0003	0.0006
34	0.3316	0.4216	0.0020	0.0032
35	0.2955	0.3852	0.0005	0.0009
36	0.4730	0.8984	7.5656 × 10^−8^	8.4735 × 10^−7^
37	0.5478	0.8015	0.0015	0.0027
38	0.9854	1.9882	7.1907 × 10^−7^	4.0268 × 10^−6^
39	0.6477	1.4941	2.3007 × 10^−8^	6.4420 × 10^−7^
G0	5.1182	3.6419	0.0057	0.0082
G1	6.9053	5.0230	0.0060	0.0085
G2	65.7748	66.3944	0.7857	0.8301
G3	15.4925	17.3621	0.0028	0.0042
G4	2.6490	5.4381	1.9742 × 10^−7^	1.7355 × 10^−6^
S0	18.0655	12.8375	0.0011	0.0021
S1	21.4848	19.4781	2.6925 × 10^−6^	1.3707 × 10^−5^
S2	42.8021	47.0417	0.0020	0.0032
S3	11.6473	13.8330	0.0001	0.0003
S4	2.1628	4.5308	2.1693 × 10^−7^	1.7355 × 10^−6^
B	5.7175	4.3939	0.0003	0.0007
CF	29.8147	21.7824	0.0002	0.0005
LB	79.2816	74.9518	4.5240 × 10^−5^	0.0001
HB	17.9680	22.9103	9.4310 × 10^−6^	3.7724 × 10^−5^
AF	3.8975	6.2130	4.1194 × 10^−6^	1.7745 × 10^−5^
HM	2.6863	2.0465	6.8624 × 10^−8^	8.4735 × 10^−7^

*Abbreviations: GP – glycan peak; q – Benjamini-Hochberg-corrected p value; G0 – agalactosylated glycans; G1-G4 – mono-, di-, tri-, tetra-galactosylated glycans. S0 – asialylated glycans; S1-S4 – mono-, di-, tri-, tetra-sialylated glycans; B – bisecting GlcNAc glycans; CF – core fucosylated glycans; LB – low-branched glycans; HB – high-branched glycans; AF – afucosylated glycans; HM – high-mannose glycans.

**Figure 1:**
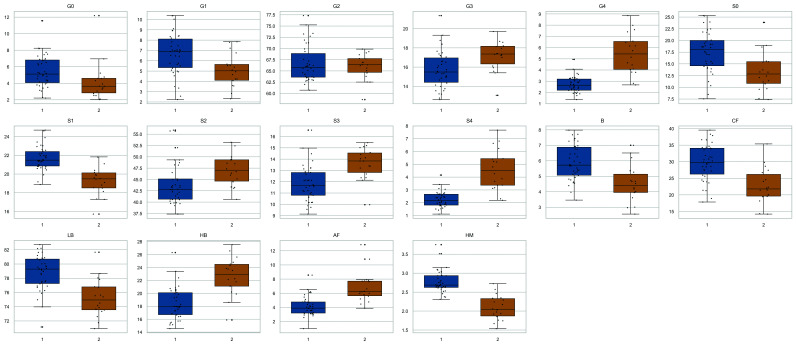
The distribution of derived plasma glycan traits in the group undergoing elective surgery (blue) and the group consisting of children with appendicitis associated with acute inflammation (orange). Each box represents the interquartile range (IQR) with the median indicated by the central line, while whiskers extend to 1.5× IQR. Individual black dots represent measurements from individual participants.

This structural shift became even more apparent for derived glycan traits. Before surgery, children with appendicitis exhibited significantly increased levels of highly galactosylated (G3, G4), highly sialylated (S2, S3, S4), antennary fucosylated (AF), and high branching (HB) structures. Conversely, they had significantly decreased glycan traits reflecting lower branching (LB), neutral structures (S0), monosialylation (S1), agalactosylation (G0), monogalactosylation (G1), core fucosylation (CF), high mannose structures (HM), and bisecting GlcNAc (B). Collectively, these findings indicate a shift toward a more complex, highly branched and highly sialylated plasma glycome in acute appendicitis (Figure 1, Table 1).

When IgG glycosylation was compared between the groups, only two peaks (IGP17 and IGP20, both decreasing in children with appendicitis) and one glycan trait (CF, increasing in children with appendicitis) showed a significant difference preoperatively (Supplemental Table 1(Supplementary Table 1)). IgG glycosylation did not significantly change before vs after surgery (Supplemental Table 2(Supplementary Table 2)). However, significant changes in 17 out of 39 plasma glycan peaks 24 hours postoperatively revealed a clear postoperative glycomic response.

At the individual peak level, GP27, GP33, GP35, and GP39 significantly increased, while GP1-GP7, GP10, GP11, GP13, GP16, GP17, and GP23 significantly decreased in the appendicitis group. These changes suggest selective remodeling of the plasma glycome 24 hours after surgery, with certain larger or more branched glycans becoming more abundant, while smaller or simpler glycans decreased.

**Figure 2:**
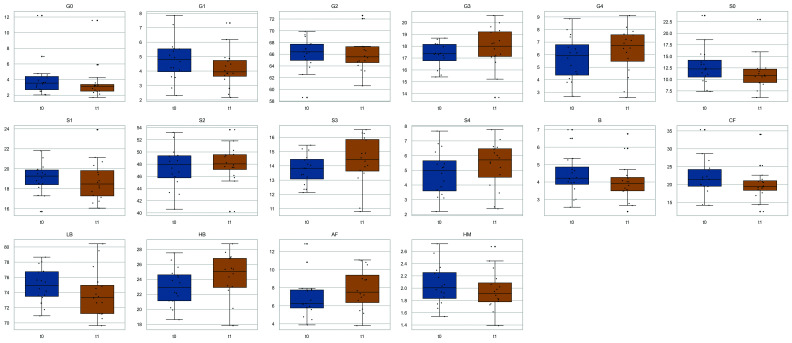
The distribution of derived plasma glycan traits in children with appendicitis at two timepoints: before surgery (blue) and 24 hours after surgery (orange). Each box indicates the interquartile range (IQR) with the median shown as the central line, and whiskers extending to 1.5× IQR. Individual black dots represent measurements from individual participants.

Derived plasma glycan traits confirmed these shifts. Postoperatively, AF significantly increased, whereas G0, G1, S0, B, and HM significantly decreased. Together, these findings reflect an acute postoperative glycomic response, highlighting dynamic remodeling of specific glycan features rather than a uniform structural shift (Figure 2, Table 2).

**Table 2:** Plasma glycan peaks (GP1-GP39) and 16 derived glycosylation traits before surgery and 24 h after surgery in children undergoing elective surgery and children with appendicitis

GP number	Minor elective surgery				Appendicitis group			
	before surgery	24 h after surgery	p	q	before surgery	24 h after surgery	p	q
1	3.4369	3.3868	0.4051	0.6968	2.4783	2.1419	0.0027	0.0107
2	1.5508	1.5536	0.0295	0.5749	1.1066	0.9967	0.0001	0.0013
3	0.0302	0.0294	0.0599	0.6379	0.0241	0.0214	0.0017	0.0078
4	3.7484	3.9404	0.2716	0.6920	2.6459	2.2755	0.0017	0.0078
5	1.5027	1.4257	0.5707	0.7794	1.0429	0.9320	0.0042	0.0138
6	0.7825	0.7900	0.1920	0.6920	0.6356	0.5296	0.0000	0.0009
7	0.9578	0.9559	0.4697	0.7108	0.7142	0.6282	0.0006	0.0054
8	1.2761	1.3004	0.8220	0.8813	1.2610	1.2396	0.8209	0.8358
9	0.0692	0.0646	0.3624	0.6920	0.0477	0.0436	0.0739	0.1380
10	4.0821	4.0920	0.3149	0.6920	2.7517	2.2987	0.0017	0.0078
11	0.5078	0.5190	0.4230	0.6968	0.4195	0.3701	0.0155	0.0377
12	1.4944	1.4679	0.0308	0.5749	1.1401	1.0321	0.0250	0.0559
13	0.5861	0.5337	0.6581	0.8190	0.3615	0.3136	0.0110	0.0280
14	13.1618	13.3031	0.5291	0.7794	13.3773	12.7523	0.7057	0.7457
15	0.3154	0.3057	0.6469	0.8190	0.3075	0.2881	0.1754	0.2455
16	5.4254	5.3738	0.1663	0.6920	3.7650	3.4809	0.0002	0.0017
17	1.1895	1.1454	0.3461	0.6920	0.8931	0.8287	3.0518 × 10^−5^	0.0009
18	4.4100	4.4209	0.5920	0.7893	3.7507	3.6499	0.3484	0.4336
19	1.0494	1.0113	0.1866	0.6920	0.7857	0.7864	0.5966	0.6682
20	28.4961	28.9067	0.4601	0.7108	35.1424	35.6620	0.5966	0.6682
21	0.5297	0.5153	0.4230	0.6968	0.5580	0.5932	0.0443	0.0954
22	3.7746	3.6783	0.1152	0.6920	3.1147	3.2217	0.3225	0.4105
23	1.1813	1.1613	0.0692	0.6379	0.8501	0.8184	0.0042	0.0138
24	1.6952	1.6326	0.8951	0.9113	1.4507	1.3648	0.1591	0.2284
25	0.3160	0.3275	0.6807	0.8287	0.4117	0.4264	0.3225	0.4105
26	1.6189	1.6256	0.6247	0.8135	1.6197	1.6252	0.4332	0.5161
27	0.6433	0.6603	0.1079	0.6920	1.0371	1.1619	0.0110	0.0280
28	0.7964	0.7953	0.5707	0.7794	0.5653	0.5472	0.8603	0.8603
29	0.2259	0.2385	0.1713	0.6920	0.3792	0.3988	0.7057	0.7457
30	5.3156	5.5476	0.3000	0.6920	5.2734	5.1000	0.8209	0.8358
31	0.4570	0.4431	0.7979	0.8761	0.4448	0.4317	0.3225	0.4105
32	1.3293	1.3614	0.0797	0.6379	1.8245	1.9686	0.0833	0.1413
33	2.2820	2.4865	0.8706	0.9028	3.4648	3.8413	0.0042	0.0138
34	0.3365	0.3469	0.2515	0.6920	0.4216	0.4503	0.0934	0.1495
35	0.2955	0.2989	0.7979	0.8761	0.3852	0.4560	0.0063	0.0196
36	0.4748	0.4972	0.5601	0.7794	0.9305	0.9999	0.1591	0.2284
37	0.5533	0.6340	0.2325	0.6920	0.8666	0.8880	0.2312	0.3158
38	1.0144	1.0993	0.1814	0.6920	2.1129	2.2261	0.1167	0.1815
39	0.6680	0.6769	0.4051	0.6968	1.5968	2.0839	0.0214	0.0499
G0	5.1182	5.0372	0.2856	0.6920	3.5404	3.1473	0.0021	0.0092
G1	6.8570	6.6829	0.3226	0.6920	4.8008	3.9432	0.0010	0.0070
G2	65.7748	66.7729	0.8341	0.8813	66.3944	65.5424	0.6685	0.7341
G3	15.5400	15.9113	0.7037	0.8345	17.3621	18.0019	0.0739	0.1380
G4	2.7271	2.9397	0.2451	0.6920	5.9376	6.7172	0.0577	0.1154
S0	17.6142	17.7298	0.3074	0.6920	12.2576	10.8044	0.0013	0.0078
S1	21.4848	21.6596	0.9197	0.9197	19.2724	18.4732	0.4332	0.5161
S2	42.8021	43.0363	0.7621	0.8710	47.9205	48.0412	0.5966	0.6682
S3	11.7069	12.0397	0.3381	0.6920	13.8330	14.4489	0.0833	0.1413
S4	2.2622	2.4436	0.2087	0.6920	4.9878	5.7066	0.0577	0.1154
B	5.6737	5.5428	0.1307	0.6920	4.2193	3.9097	0.0001	0.0011
CF	29.6164	29.1233	0.2648	0.6920	21.4604	19.4545	0.0010	0.0070
LB	79.2078	78.3854	0.4697	0.7108	74.9518	73.3653	0.0934	0.1495
HB	17.9741	18.9282	0.3707	0.6920	22.9103	25.0469	0.0833	0.1413
AF	3.8975	4.1242	0.7152	0.8345	6.2417	7.5072	0.0076	0.0214
HM	2.6937	2.6551	0.0516	0.6379	2.0051	1.9152	0.0076	0.0214

*Abbreviations: GP – glycan peak; q – Benjamini-Hochberg corrected p value; G0 – agalactosylated glycans; G1-G4 – mono-, di-, tri-, tetra-galactosylated glycans; S0 – asialylated glycans; S1-S4 – mono-, di-, tri-, tetra-sialylated glycans; B – bisecting GlcNAc glycans; CF – core fucosylated glycans; LB – low-branched glycans; HB – high-branched glycans; AF – afucosylated glycans; HM – high-mannose glycans.

## Discussion

In this study, acute appendicitis was accompanied by substantial remodeling of the plasma N-glycome characterized by increased structural complexity, branching, and sialylation. On the other hand, mild postoperative inflammation following minor surgery did not induce detectable glycomic changes in plasma N-glycome and IgG N-glycome within the first 24 hours. These results indicate that glycosylation changes in children primarily reflect the intensity of systemic inflammation rather than surgical intervention itself.

Our study revealed extensive preoperative differences across multiple derived glycan traits, indicating pronounced glycomic remodeling associated with severe acute inflammation. Compared with the minor surgery group, children with appendicitis exhibited elevated CRP concentrations accompanied by coordinated increases in highly branched glycans (G3, G4, HB), highly sialylated structures (S2-S4), and AF. In contrast, traits reflecting lower structural complexity (LB, S0, S1, G0, G1, CF, B, and HM) were decreased.

The increase in tri- and tetra-antennary glycans represents a characteristic feature of inflammation-associated plasma glycosylation (29). Highly branched glycans are predominantly carried by acute-phase glycoproteins synthesized in the liver, such as α1-acid glycoprotein, haptoglobin, and α2-macroglobulin, whose production is markedly upregulated during systemic inflammatory responses (29). Enhanced branching expands the number of available antennae for terminal modifications and is linked to increased activity of N-acetylglucosaminyltransferases induced under inflammatory conditions (29,40).

Consistent with this structural expansion, appendicitis was associated with higher levels of di-, tri-, and tetra-sialylated glycans (S2-S4). Increased sialylation is a feature of the acute-phase response and contributes to the prolonged circulatory half-life of plasma proteins by preventing hepatic clearance via the asialoglycoprotein receptor (12,41). The simultaneous rise in antennary fucosylation further supports the activation of inflammation-related glycosylation pathways, as antennary fucose residues are involved in immune cell interactions and inflammatory signaling processes (22).

Conversely, reductions in G0, G1, S0, and bisecting GlcNAc traits compared with the minor surgery group suggest a diminished contribution of glycoproteins characterized by less extensively processed glycans, alongside increased dominance of highly glycosylated acute-phase proteins. Decreased core fucosylation and high-mannose structures further indicate a shift in the overall plasma protein composition during severe inflammation rather than isolated modification of individual glycans.

Importantly, because the reference group also experienced surgical intervention but exhibited no comparable glycomic alterations, these findings demonstrate that the observed trait changes are not attributable to surgery itself but reflect differences in inflammatory burden. The coordinated directionality across multiple glycan traits, therefore, defines a systemic inflammatory glycan phenotype associated with acute appendicitis.

Despite biochemical evidence of an inflammatory response following minor surgery, reflected by increased CRP concentrations, plasma N-glycan composition did not significantly change. This finding indicates that mild or transient postoperative inflammation is insufficient to induce detectable systemic plasma glycomic remodeling within the first 24 hours. Although acute inflammatory responses are known to influence glycosylation, the absence of plasma glycome changes in children undergoing elective surgery suggests the existence of an inflammatory threshold below which systemic alterations in plasma protein glycosylation do not occur.

In contrast, significant longitudinal alterations in the plasma N-glycome were detected exclusively in children with acute appendicitis. Postoperative changes were characterized by increased AF, accompanied by decreases in G0, G1, S0, B, CF, and HM. These changes likely reflect the activation of the acute-phase response, during which glycoforms associated with inflammation-related plasma proteins increase in relative abundance, while glycans characteristic of constitutive plasma proteins decline. Similar inflammation-associated remodeling of the total plasma N-glycome has been reported following major abdominal surgery, where glycans of lower structural complexity consistently decreased postoperatively (31). Collectively, these findings indicate that glycomic restructuring represents a response to substantial systemic inflammation rather than a general consequence of surgical intervention and may also capture early resolution of inflammation following appendectomy.

In contrast to plasma glycosylation, IgG N-glycosylation remained stable between preoperative and postoperative samples in both study groups. IgG glycosylation represents an integrated molecular phenotype characterized by substantial physiological stability, reflecting cumulative regulation of immune and metabolic processes rather than rapid short-term fluctuations (15,17). This stability is consistent with the biological kinetics of IgG, which exhibits a relatively long circulatory half-life of approximately 21 days, with subclass-specific variation among IgG isoforms (42). Because IgG glycosylation primarily reflects the properties of newly synthesized antibodies rather than modification of circulating molecules, measurable changes are unlikely to occur within a 24-hour observation period despite ongoing immune activation.

The plasma glycome dynamics observed in appendicitis may be explained by two non-mutually exclusive mechanisms. First, inflammatory signaling may actively regulate hepatic glycosyltransferase activity, leading to altered glycosylation of newly synthesized acute-phase proteins. Because liver-derived plasma proteins respond rapidly to inflammatory stimuli, their glycosylation patterns can change over short time intervals. Alternatively, the observed shifts may primarily reflect alterations in plasma protein composition. Acute inflammation is accompanied by rapid turnover of circulating proteins, including increased synthesis of acute-phase glycoproteins and reduced relative abundance of constitutive proteins such as albumin and immunoglobulins, thereby reshaping the composite plasma glycome. As total plasma N-glycome analysis represents an aggregate signal derived from multiple glycoproteins, the present study cannot mechanistically distinguish between these processes. It is however likely that both inflammation-driven regulation of glycosylation and changes in plasma protein composition contribute to the temporal glycomic signature observed following appendectomy.

This study has several limitations. The relatively small sample size and single-center design may limit statistical power and the generalizability of findings to broader pediatric populations. Postoperative glycomic changes were assessed within a 24-hour window, which may not capture the full trajectory of glycome remodeling during inflammation resolution, and longer follow-up would be needed to characterize time-dependent dynamics more completely. Furthermore, the absence of healthy age-matched controls limits the ability to define a physiological reference range for pediatric plasma N-glycosylation. Finally, clinical heterogeneity in disease severity and stage at presentation among children with appendicitis may have introduced biological variability within the group, potentially obscuring glycomic associations with specific clinical features.

Overall, these findings support the concept that plasma N-glycosylation serves as an integrative molecular readout of systemic inflammatory burden rather than a universal response to surgical stress. The results extend the current understanding of inflammation-associated glycosylation in pediatric populations and highlight plasma glycome profiling as a promising systems-level biomarker for assessing inflammatory processes and disease severity in children.

## References

[1] Reily C Stewart TJ Renfrow MB Novak J Glycosylation in health and disease. Nat Rev Nephrol 2019 15 346 66 10.1038/s41581-019-0129-4 30858582 PMC6590709

[2] Varki A, Cummings RD, Esko JD, Stanley P, Hart GW, Aebi M, et al. Essentials of Glycobiology [Internet]. Cold Spring Harbor (NY). 2022;892. 35536922

[3] Gornik O Lauc G Glycosylation of serum proteins in inflammatory diseases. Dis Markers 2008 25 267 78 10.1155/2008/493289 19126970 PMC3827815

[4] Apweiler R Hermjakob H Sharon N On the frequency of protein glycosylation, as deduced from analysis of the SWISS-PROT database. Biochim Biophys Acta 1999 1473 4 8 10.1016/S0304-4165(99)00165-8 10580125

[5] Lauc G Rudan I Campbell H Rudd PM Complex genetic regulation of protein glycosylation. Mol Biosyst 2010 6 329 35 10.1039/B910377E 20094651

[6] Moremen KW Tiemeyer M Nairn AV Vertebrate protein glycosylation: diversity, synthesis and function. Nat Rev Mol Cell Biol 2012 13 448 62 10.1038/nrm3383 22722607 PMC3934011

[7] Marth JD Grewal PK Mammalian glycosylation in immunity. Nat Rev Immunol 2008 8 874 87 10.1038/nri2417 18846099 PMC2768770

[8] Sakabe K Wang Z Hart GW Beta-N-acetylglucosamine (O-GlcNAc) is part of the histone code. Proc Natl Acad Sci U S A 2010 107 19915 20 10.1073/pnas.1009023107 21045127 PMC2993388

[9] Alavi A Axford JS Sweet and sour: the impact of sugars on disease. Rheumatology (Oxford) 2008 47 760 70 10.1093/rheumatology/ken081 18375404

[10] Gornik O Pavić T Lauc G Alternative glycosylation modulates function of IgG and other proteins - implications on evolution and disease. Biochim Biophys Acta 2012 1820 1318 26 10.1016/j.bbagen.2011.12.004 22183029

[11] van de Bovenkamp FS Hafkenscheid L Rispens T Rombouts Y The Emerging Importance of IgG Fab Glycosylation in Immunity. J Immunol 2016 196 1435 41 10.4049/jimmunol.1502136 26851295

[12] Radovani B Gudelj I N-Glycosylation and Inflammation; the Not-So-Sweet Relation. Front Immunol 2022 ••• 13 35833138 10.3389/fimmu.2022.893365 PMC9272703

[13] Quast I Peschke B Lünemann JD Regulation of antibody effector functions through IgG Fc N-glycosylation. Cell Mol Life Sci 2017 74 837 47 10.1007/s00018-016-2366-z 27639381 PMC11107549

[14] Pučić M Knežević A Vidič J Adamczyk B Novokmet M Polašek O High throughput isolation and glycosylation analysis of IgG-variability and heritability of the IgG glycome in three isolated human populations. Mol Cell Proteomics 2011 10 10.1074/mcp.M111.010090 21653738 PMC3205872

[15] Štambuk J Nakić N Vučković F Pučić-Baković M Razdorov G Trbojević-Akmačić I Global variability of the human IgG glycome. Aging (Albany NY) 2020 12 1 13 10.18632/aging.103884 32788422 PMC7467356

[16] Knežević A Polašek O Gornik O Rudan I Campbell H Hayward C Variability, heritability and environmental determinants of human plasma N-glycome. J Proteome Res 2009 8 694 701 10.1021/pr800737u 19035662

[17] Rapčan B Song M Frkatović-Hodžić A Pribić T Vuk J Beletić A Glycan clock of ageing-analytical precision and time-dependent inter- and i-individual variability. Geroscience 2024 46 5781 96 10.1007/s11357-024-01239-4 38877341 PMC11494675

[18] Knežević A Gornik O Polašek O Pučić M Redžić I Novokmet M Effects of aging, body mass index, plasma lipid profiles, and smoking on human plasma N-glycans. Glycobiology 2010 20 959 69 10.1093/glycob/cwq051 20356825

[19] Trbojević-Akmačić I Lageveen-Kammeijer GSM Heijs B Petrović T Deriš H Wuhrer M High-Throughput Glycomic Methods. Chem Rev 2022 122 15865 913 10.1021/acs.chemrev.1c01031 35797639 PMC9614987

[20] Gudelj I Lauc G Pezer M Immunoglobulin G glycosylation in aging and diseases. Cell Immunol 2018 333 65 79 10.1016/j.cellimm.2018.07.009 30107893

[21] Pongracz T Mayboroda OA Wuhrer M The Human Blood N-Glycome: Unraveling Disease Glycosylation Patterns. JACS Au 2024 4 1696 708 10.1021/jacsau.4c00043 38818049 PMC11134357

[22] Kissel T Toes REM Huizinga TWJ Wuhrer M Glycobiology of rheumatic diseases. Nat Rev Rheumatol 2023 19 28 43 10.1038/s41584-022-00867-4 36418483 PMC9684870

[23] Pučić M Mužinić A Novokmet M Škledar M Pivac N Lauc G Changes in plasma and IgG N-glycome during childhood and adolescence. Glycobiology 2012 22 975 82 10.1093/glycob/cws062 22426998

[24] Raynor A Haouari W Lebredonchel E Foulquier F Fenaille F Bruneel A Biochemical diagnosis of congenital disorders of glycosylation. Adv Clin Chem 2024 120 1 43 38762238 10.1016/bs.acc.2024.03.001

[25] Cheng HD Tirosh I de Haan N Stöckmann H Adamczyk B McManus CA IgG Fc glycosylation as an axis of humoral immunity in childhood. J Allergy Clin Immunol 2020 145 710 713.e9 10.1016/j.jaci.2019.10.012 31669096 PMC7010538

[26] Rudman N Kifer D Kaur S Simunović V Cvetko A Pociot F Children at onset of type 1 diabetes show altered N-glycosylation of plasma proteins and IgG. Diabetologia 2022 65 1315 27 10.1007/s00125-022-05703-8 35622127 PMC9283363

[27] Kvist M Välimaa L Harel A Malmi S Tuomisto A Glycans as Potential Diagnostic Markers of Traumatic Brain Injury in Children. Diagnostics (Basel) 2023 13 10.3390/diagnostics13132181 37443575 PMC10340482

[28] Gabay C Kushner I Acute-phase proteins and other systemic responses to inflammation. N Engl J Med 1999 340 448 54 10.1056/NEJM199902113400607 9971870

[29] Clerc F Reiding KR Jansen BC Kammeijer GSM Bondt A Wuhrer M Human plasma protein N-glycosylation. Glycoconj J 2015 33 309 10.1007/s10719-015-9626-2 26555091 PMC4891372

[30] Novokmet M Lukić E Vučković F Durić Ž Keser T Rajšl K Changes in IgG and total plasma protein glycomes in acute systemic inflammation. Sci Rep 2014 ••• 4 10.1038/srep04347 24614541 PMC3949295

[31] Gudelj I Baciarello M Ugrina I De Gregori M Napolioni V Ingelmo PM Changes in total plasma and serum N-glycome composition and patient-controlled analgesia after major abdominal surgery. Sci Rep 2016 ••• 6 10.1038/srep31234 27501865 PMC4977520

[32] Gornik O Gornik I Gašparović V Lauc G Change in transferrin sialylation is a potential prognostic marker for severity of acute pancreatitis. Clin Biochem 2008 41 504 10 10.1016/j.clinbiochem.2008.01.026 18307987

[33] Gornik O Gornik I Kolednjak IZ Lauc G Change of transferrin sialylation differs between mild sepsis and severe sepsis and septic shock. Intern Med 2011 50 861 9 10.2169/internalmedicine.50.4704 21498934

[34] Simon AK, Hollander GA, McMichael A. Evolution of the immune system in humans from infancy to old age. Proc Biol Sci. 2015 Dec 23;282(1821). 10.1098/rspb.2014.3085 PMC4707740 26702035

[35] Clinical Laboratory Diagnostics [Internet].

[36] Bernardi L Bossù G Dal Canto G Giannì G Esposito S Biomarkers for Serious Bacterial Infections in Febrile Children. Biomolecules 2024 14 10.3390/biom14010097 38254697 PMC10813546

[37] Hanić M Lauc G Trbojević-Akmačić I N-Glycan Analysis by Ultra-Performance Liquid Chromatography and Capillary Gel Electrophoresis with Fluorescent Labeling. Curr Protoc Protein Sci 2019 97 10.1002/cpps.95 31517449

[38] Pučić M Knežević A Vidič J Adamczyk B Novokmet M Polašek O High throughput isolation and glycosylation analysis of IgG-variability and heritability of the IgG glycome in three isolated human populations. Mol Cell Proteomics 2011 10 10.1074/mcp.M111.010090 21653738 PMC3205872

[39] Zaytseva OO Freidin MB Keser T Štambuk J Ugrina I Šimurina M Heritability of Human Plasma N-Glycome. J Proteome Res 2020 19 85 91 10.1021/acs.jproteome.9b00348 31747749

[40] Dennis JW Nabi IR Demetriou M Metabolism, cell surface organization, and disease. Cell 2009 139 1229 41 10.1016/j.cell.2009.12.008 20064370 PMC3065826

[41] Zhu W, Zhou Y, Guo L, Feng S. Biological function of sialic acid and sialylation in human health and disease. Cell Death Discovery 2024 10:1. 2024 Sep 30;10(1):415-. 10.1038/s41420-024-02180-3 PMC11442784 39349440

[42] Napodano C Marino MP Stefanile A Pocino K Scatena R Gulli F Immunological Role of IgG Subclasses. Immunol Invest 2021 50 1 18 10.1080/08820139.2020.1775643 32522062

