# Supplementary material for: Perioperative changes in IgG and plasma N-glycosylation in children with acute appendicitis and elective surgery: a prospective study
Source: Croat Med J. 2026 Jun;67(3):164–75. doi: 10.3325/cmj.2026.67.164 (PMC13247734; doi:10.3325/cmj.2026.67.164)
Supplement: Supplementary Figure 2 [file CroatMedJ_67_s010.pdf]

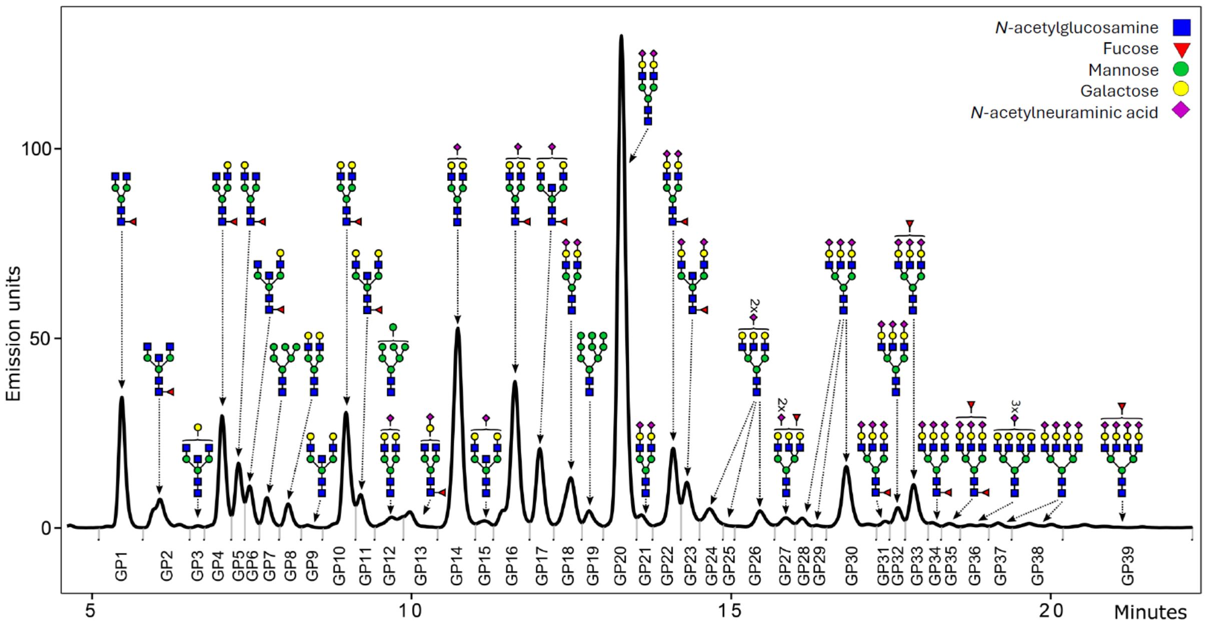

Supplementary Figure 2. Annotated and integrated chromatogram of total plasma N-glycans separated by HILIC-UHPLC analysis. Raw glycan abundances are computed as areas under the curve of corresponding chromatographic peak.
