# Supplementary material for: Perioperative changes in IgG and plasma N-glycosylation in children with acute appendicitis and elective surgery: a prospective study
Source: Croat Med J. 2026 Jun;67(3):164–75. doi: 10.3325/cmj.2026.67.164 (PMC13247734; doi:10.3325/cmj.2026.67.164)
Supplement: Supplementary Table 1 [file CroatMedJ_67_s011.pdf]

Supplementary Table 1. Comparison of IgG glycan peaks (IGP1–IGP24) and derived glycosylation traits between the control group of children undergoing elective surgery and children with appendicitis. Values represent median normalized abundances for each group. Statistical significance was assessed using the p-value, and q-values represent false discovery rate (FDR)–adjusted p-values.

IGP number	median 1	median 2	p	q
1	0,1007	0,0999	0,9355	0,9613
2	0,2367	0,2133	0,9613	0,9613
3	0,1125	0,1202	0,2643	0,4845
4	20,9728	20,4215	0,8334	0,9484
5	0,2469	0,1847	0,0225	0,0826
6	3,4951	3,2856	0,7340	0,9317
7	0,1634	0,1604	0,7957	0,9378
8	20,6450	21,3041	0,3564	0,5236
9	8,2920	9,2089	0,0675	0,1857
10	3,2524	3,5635	0,0956	0,2427
11	0,3615	0,4029	0,0207	0,0826
12	0,4026	0,4195	0,7957	0,9378
13	0,1769	0,1860	0,3480	0,5236
14	16,1643	15,9418	0,5281	0,7261
15	1,3501	1,4251	0,1641	0,3868
16	2,7481	2,9635	0,2930	0,5088
**17**	**1,2296**	**0,8576**	**$4{,}2526 \times 10^{-5}$**	**0,0007**
18	12,0296	11,0175	0,3649	0,5236

19	1,3920	1,4345	0,6274	0,8282
**20**	**0,5136**	**0,3054**	**0,0007**	**0,0075**
21	1,3320	0,7654	0,0116	0,0638
22	0,1695	0,1462	0,0503	0,1508
23	2,5257	2,2807	0,0466	0,1508
24	1,5014	1,5227	0,9484	0,9613
G0	24,6975	24,2441	0,9613	0,9613
G1	35,7079	38,2252	0,0080	0,0526
G2	38,8858	36,7427	0,2128	0,4488
S	23,0830	21,8638	0,2312	0,4488
S1	17,7798	16,5966	0,3564	0,5236
S2	5,7174	4,9801	0,0216	0,0826
B	11,9792	12,3964	0,2250	0,4488
**CF**	**95,3532**	**96,1225**	**0,0057**	**0,0467**

IGP- IgG glycan peak, median 1 – median of group 1 (elective surgery), median 2 – median of group 2 (appendicitis), p – calculated p value, q– Benjamini-Hochberg corrected p value
