# Supplementary material for: Perioperative changes in IgG and plasma N-glycosylation in children with acute appendicitis and elective surgery: a prospective study
Source: Croat Med J. 2026 Jun;67(3):164–75. doi: 10.3325/cmj.2026.67.164 (PMC13247734; doi:10.3325/cmj.2026.67.164)
Supplement: Supplementary Table 2 [file CroatMedJ_67_s012.pdf]

Supplementary Table 2. Within-group comparison of IgG glycan peaks (IGP1–IGP24) and eight derived glycosylation traits between t0 (before surgery) and t1 (24 h after surgery) in Group 1 (children undergoing elective surgery) and Group 2 (children with appendicitis). Values represent median normalized abundances at each timepoint. p-values indicate nominal statistical significance for the t0–t1 comparison within each group, while q-values represent p-values adjusted for multiple testing using the Benjamini–Hochberg false discovery rate (FDR) correction.

IGP number	Group 1 between operation difference				Group 2 between operation difference			
	median 1	median 2	p	q	median 1	median 2	p	q
1	0,1054	0,0970	0,8754	0,9926	0,1007	0,0972	0,6794	0,9342
2	0,2151	0,2394	0,2951	0,8617	0,2152	0,2228	0,8288	0,9341
3	0,1129	0,1146	0,3309	0,8617	0,1301	0,1284	0,6507	0,9342
4	21,2184	21,0426	0,5361	0,8617	20,4215	20,4880	0,7680	0,9387
5	0,2488	0,2452	0,9926	0,9926	0,1767	0,1998	0,5412	0,9342
6	3,6081	3,5699	0,9779	0,9926	3,2856	3,2154	0,1336	0,9342
7	0,1546	0,1521	0,9192	0,9926	0,1647	0,1654	0,1819	0,9342
8	20,7854	21,1714	0,5361	0,8617	21,1211	21,5594	0,6226	0,9342
9	8,2593	8,2305	0,7053	0,9491	8,6355	8,6597	0,1134	0,9342
10	3,2716	3,2816	0,2169	0,8617	3,5635	3,5629	0,4653	0,9342
11	0,3616	0,3591	0,5361	0,8617	0,4029	0,4076	0,1042	0,9342
12	0,3967	0,3955	0,8609	0,9926	0,4591	0,4635	0,5949	0,9342
13	0,1754	0,1753	0,2699	0,8617	0,1862	0,1935	0,3955	0,9342

14	16,1123	16,0238	0,9485	0,9926	15,9418	15,6951	0,1688	0,9342
15	1,3731	1,3322	0,5000	0,8617	1,4251	1,4485	1,0000	1,0000
16	2,7219	2,6850	0,7053	0,9491	2,9635	2,8737	0,4180	0,9342
17	1,2461	1,1724	0,4105	0,8617	0,8540	0,9224	0,5153	0,9342
18	11,9585	11,8053	0,3498	0,8617	11,0175	10,9166	0,6507	0,9342
19	1,3862	1,3348	0,7190	0,9491	1,4264	1,4605	0,9843	1,0000
20	0,5174	0,4720	0,3896	0,8617	0,2816	0,3437	0,6226	0,9342
21	1,3527	1,2613	0,5239	0,8617	0,7383	0,9150	0,5153	0,9342
22	0,1662	0,1567	0,0908	0,8617	0,1424	0,1493	0,2935	0,9342
23	2,5151	2,4898	0,8754	0,9926	2,2612	2,4099	0,3525	0,9342
24	1,5032	1,5512	0,9046	0,9926	1,5093	1,4860	0,9530	1,0000
G0	24,8760	25,2391	0,5483	0,8617	25,0687	25,0853	0,8288	0,9431
G1	35,7488	35,7423	0,4000	0,8617	37,6431	38,1133	0,6507	0,9342
G2	38,7777	38,4411	0,4883	0,8617	36,0436	36,1610	0,8906	0,9796
S	23,0005	22,6532	0,4653	0,8617	21,5828	22,1631	0,6794	0,9342
S1	17,6460	17,1038	0,3217	0,8617	16,5966	16,8313	0,3736	0,9342
S2	5,7325	5,4497	0,6117	0,9175	4,8556	4,9628	0,7680	0,9387
B	12,0018	11,6495	0,3794	0,8617	12,3964	12,1867	0,7381	0,9387
CF	95,3665	95,4022	0,5239	0,8617	96,1225	96,0648	0,5412	0,9342

IGP- IgG glycan peak, median 1 – median of group 1 (elective surgery), median 2 – median of group 2 (appendicitis), p – calculated p value, q– Benjamini-Hochberg corrected p value
